# Prevalence of Depression Among Older Adults Visiting the Primary Healthcare Centers in Jizan City, Saudi Arabia: An Analytical Cross-Sectional Study

**DOI:** 10.7759/cureus.52847

**Published:** 2024-01-24

**Authors:** Faizah Alfaifi, Mona Elmahdy, Maged A El-Setouhy, Abdulrahman Alfaifi

**Affiliations:** 1 Preventive Medicine, Jazan Health Affairs, Ministry of Health, Jazan, SAU; 2 Family and Community Medicine, Faculty of Medicine, Jazan University, Jazan, SAU

**Keywords:** saudi, depression, aging, geriatric, older adult, elderly

## Abstract

Background and objective

Older adults are susceptible to various disorders, including depression. Depression manifests as a disorder characterized by a persistent depressed mood, feelings of sadness and loneliness, sleep disturbances, and loss of interest lasting for two weeks or more. This condition can significantly affect the quality of life for older adults, resulting in adverse outcomes that have a negative impact on their overall well-being. In this study, our objective was to assess the prevalence of depression and identify associated risk factors among individuals aged 60 years and above who attended Primary Healthcare Centers (PHCCs) in Jizan City.

Methodology

We conducted an analytical cross-sectional study among older people attending PHCCs in Jizan City between May and December 2022. In this study, we interviewed 300 participants using the Patient Health Questionnaire-9 (PHQ-9) to assess depression. IBM SPSS Statistics for Windows, Version 25.0 (IBM Corp., Armonk, NY) was used for data entry and analysis. We used the chi-square test and binary logistic regression model to detect the associations and predictors of depression among older adults.

Results

One-third of the study participants experienced depression, with 49 (16%) exhibiting mild depression, 33 (11%) reporting moderate depression, and 17 (6%) individuals indicating moderately severe depression. Depression was more common among females (158, 52.7%) than males (142, 47.3%). The predictors of depression among the study participants were the dependency to perform daily tasks and abnormal sleep duration.

Conclusions

Depression is a prevalent health problem among older people in Jizan City, particularly among females, those who are divorced or widowed, individuals dependent on others for daily tasks, and those with abnormal sleep duration. We conclude that we need more research on older adults to assess their mental problems and fill in the literature gap.

## Introduction

Aging is a biological fact with its own dynamics. The concept of aging varies between societies. It is defined as deteriorating physiological function necessary for survival and reproduction over time [1]. Healthy aging is the ability of aged individuals to do their daily activities independently and maintain their physical, psychological, mental, and social well-being [2]. In developed countries, chronological time is indicative of aging, where the age range from 60 to 65 typically signifies retirement and marks the onset of old age. In other countries, age-related disability in performing tasks or ineffective participation in society means the beginning of old age [3].

Recently, there has been an increase in the number of older people globally. The life expectancy of the general population increased from 66.4 to 73.4 years between 2000 and 2019 [4]. During 2020, the number of older adults worldwide was one billion, which will double by the end of 2050 [5]. This increase might be attributed to the better quality of life nowadays. In Saudi Arabia, the latest statistics indicate that approximately 5% of the total population is older adults [6].

In alignment with the 2030 Saudi vision, the Kingdom of Saudi Arabia is dedicated to improving the quality of life for the people. Moreover, Saudi Vision aims to improve the overall life expectancy of the population, with a goal to increase it from 74 to 80 years by the end of 2030 [7].

Many factors affect older adults' health, like aging, loneliness, disabilities, chronic diseases, and losing loved ones [8]. These factors lead to several mental health problems, such as depression, anxiety, stress, and dementia. Depression is the most familiar mental disease affecting this age group. According to the World Health Organization (WHO), 7% of older adults worldwide are affected by depression [9].

This issue in aged people is often misdiagnosed and underreported due to the misconception that depression is a normal part of aging [10]. Also, the stigma of depression among older adults is another barrier to seeking treatment [11]. Unfortunately, depressed patients are more prone to suicidal attempts and self-harm and experience poorer health and quality of life [12,13]. Therefore, treatment and screening for depression in older adults is important [14].

One Saudi national survey among older adults 30 years ago showed that 39% of participants had depressive symptoms [15]. Recently, the Saudi National Mental Health Survey (SNMHS) illustrated that major depressive disorder is the third most common mental health condition in Saudi Arabia, with a diagnosis observed in 3% of males and 9% of females at some point in their lives. However, this survey did not include the Jazan and Najran regions in their sample size due to armed conflict on the southern border during the survey period. Moreover, those above 65 years were not included in that survey [16,17]. Moreover, no previous research was done in the Jazan region to assess depression among older people.

Jazan City is the capital of Jazan province, located in the southwest of Saudi Arabia adjacent to Yemen. According to the 2019 Saudi population census, the total population of the Jazan region was 1,637,361; older people constitute 7% of them [18].

We hypothesize that depression increases with advancing age. Therefore, we conducted this study to measure the prevalence of depression and its associated risk factors among the geriatric population visiting primary healthcare centers (PHCCs) in Jizan City. By the end of this study, we will better understand this problem and provide decision-makers with evidence to help fill the gap in the literature.

## Materials and methods

Design, setting, and participants

We conducted an analytical cross-sectional study at five chosen PHCCs in Jizan City, Saudi Arabia, between May and December 2022. The chosen centers were the most frequently visited PHCCs by older adults. We collected all eligible individuals presented to the chosen PHCCs within the study period.

This study recruited all individuals 60 years and above who visited the family medicine clinics and agreed to participate. We ended up with 312 eligible individuals; 12 refused to be enrolled in this study. Older people with communication difficulties were excluded.

Data collection

We collected our data through an interview questionnaire. It had two main sections. We established the first section to collect the participants' sociodemographic characteristics such as age, gender, marital status, nationality, level of education, occupation, income, presence of chronic diseases, sleeping duration, ability to perform daily tasks, number of people living with them in the same house, smoking status, and khat chewing habit.

The second section of the questionnaire assessed the depressive symptoms among study participants using the validated Arabic version of the Patient Health Questionnaire-9 (PHQ-9) after obtaining permission to use it [19,20]. This questionnaire consisted of nine questions, each with four answers to measure the frequency of the symptoms, where 0 = not present at all, 1 = several days, 2 = more than half the days, and 3 = nearly every day. The total score ranged from 0 to 27, with scores interpreted as follows: less than 5 indicates no depression, (5-9) indicates mild depression, (10-14) indicates moderate depression, (15-19) indicates moderate-to-severe depression, and (20-27) indicates severe depression. Although there are different tools to assess depression among older people, such as the Geriatric Depression Scale-15 (GDS-15), we used PHQ-9 because of its relative brevity and ease of use, which makes it attractive compared to the longer GDS-15 [21]. Then, two trained enumerators interviewed the participants to collect the questionnaires in three to five minutes. Those who could read and write were given the questionnaire to fill out by themselves with assistance as needed, while those who could not read or write were assisted in completing the questionnaire.

Data analysis

We analyzed our data using IBM SPSS Statistics for Windows, Version 25.0 (IBM Corp., Armonk, NY). We used both descriptive and inferential statistics for data analysis. At first, we used simple tabulation frequencies to give a general overview of the data. Then, we transformed PHQ-9 scores into binary outcomes: *depressed* for those who scored five and above, and *not depressed* for those below. We used the chi-square test to measure the association between the dependent and independent categorical variables. After that, all variables were included in multivariate analysis using binary logistic regression. The regression model was derived through simultaneous entry analysis. We check the fitness of this model using the Hosmer-Lemeshow test. A *P*-value of 0.05 or less was determined as the cutoff level of statistical significance.

Ethical considerations

The study was conducted by the Declaration of Helsinki and approved by the Research Ethics Committee of the Ministry of Health in Jazan (no. 2135 on May 28, 2021). Informed consent was obtained from all subjects involved in the study. All data were kept anonymous, and confidentiality was maintained.

## Results

Sociodemographic characteristics

**Figure 1:**
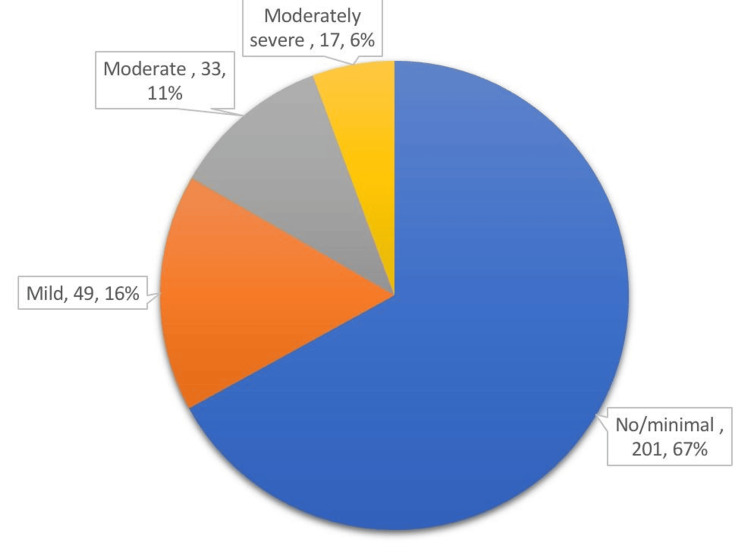
Prevalence of depression among study participants.

The response rate for the survey was 96.1% (300/312). The prevalence of depression among older adults was 33% (Figure 1).

Percentage of depression among the study sample

A total of 201 (67%) individuals had no/minimal depression, 49 (16%) had mild depression, 33 (11%) had moderate depression, and 17 (6%) had moderately severe depression. As shown in Table 1, the number of males was almost equal to that of females. The majority of the study participants (268, 89.3%) were Saudi and 32 (10.7%) were non-Saudi. The marital status distribution showed that 183 (61%) were married, 77 (25.7%) were widowed, 30 (10%) were single, and 10 (3.3%) were divorced. About 142 (47.3%) participants were illiterate, 272 (90.7%) had chronic diseases, 32 (10.7%) depended on others to perform daily tasks, and 128 (43.4%) were affected by sleeping problems. The prevalence of previous smokers was 68 (22.7%), while that of current smokers was 34 (11.3%). Regarding khat chewing, the prevalence of previous and current khat chewers was 48 (16%) for both (Table 1). 

**Table 1:** Demographic and clinical characteristics of study participants. ^a^Categories might not add up to the total number due to missing data. *n*, the number of those who answered the related variable question; IQR, interquartile range; SAR, Saudi riyal

Variables	Frequency (%)^a^
Age (*n *= 300); median (IQR)	66 (8 years)
Gender (*n *= 300)	
Male	142 (47.3)
Female	158 (52.7)
Nationality (*n *= 300)	
Saudi	268 (89.3)
Non-Saudi	32 (10.7)
Marital status (*n *= 300)	
Single	30 (10)
Married	183 (61)
Divorced	10 (3.3)
Widowed	77 (25.7)
Residence (*n* = 300)	
Urban	289 (96.3)
Rural	11 (3.7)
Living condition (296)	
Living alone	21 (7.1)
Living with others	275 (92.9)
Education level (*n *= 300)	
Illiterate	142 (47.3)
Primary school	60 (20)
Intermediate school	28 (9.3)
Secondary school	20 (6.7)
University	20 (6.7)
Postgraduate	6 (2)
Diploma	24 (8)
Employment (*n *= 300)	
Unemployed	229 (76.3)
Employed	71 (23.7)
Monthly income, SAR (*n *= 296)	
Low income, <5,000	198 (66.9)
Moderate income, 5,000-15,000	79 (26.7)
High income, >15,000	19 (6.4)
Presence of chronic disease (*n *= 300)	
Yes	272 (90.7)
No	28 (9.3)
Ability to perform daily tasks (*n* = 300)	
Dependent	32 (10.7)
Independent	268 (89.3)
Sleep duration (*n *= 295)	
Abnormal sleep duration	128 (43.4)
Normal sleep duration	167 (56.6)
Smoking status (300)	
Previous smokers	
Yes	68 (22.7)
No	232 (77.3)
Current smokers	
Yes	34 (11.3)
No	266 (88.7)
Khat chewing status (*n* = 296)	
Previous khat chewers	
Yes	48 (16)
No	248 (82.7)
Current khat chewers	
Yes	48 (16)
No	248 (82.7)

Determinants of geriatric depression

Sociodemographic variables, such as gender and marital status, were found to be associated with depression in the elderly population. The prevalence of depression among elderly females was 41.1% (65), compared to 23.9% (34) in males (*P* < 0.01). Furthermore, the prevalence of depression differed according to variations in marital status; it was lower among married (50, 27.3%) and higher among other categories (*P* < 0.05). Dependency to perform daily tasks and abnormal sleep duration were highly associated with depression (*P* < 0.001) (Table 2). 

**Table 2:** The association between depression and independent variables among the study participants. ^*^Statistical significant result: *P*-value ≤ 0.05. IQR, interquartile range; PHQ-9, Patient Health Questionnaire-9

Variables	Depression		Chi-square	*P*-value
	Yes, PHQ-9 ≥ 5 (*n *= 99), *n* (%)	No, <5 (*n *= 201), *n* (%)		
Gender			10.00	<0.01*
Male	34 (23.9)	108 (76.1)		
Female	65 (41.1)	93 (58.9)		
Nationality			0.05	0.82
Saudi	89 (33.2)	179 (66.8)		
Non-Saudi	10 (31.3)	22 (68.8)		
Marital status			6.91	0.03*
Widowed/divorced	37 (42.5)	50 (57.5)		
Married	50 (27.3)	133 (72.7)		
Single	12 (40)	18 (60)		
Residence			0.80	0.37
Urban	94 (32.5)	195 (67.5)		
Rural	5 (45.5)	6 (54.5)		
Living condition			0.27	0.61
Living alone	8 (38.1)	13 (61.9)		
Living with others	91 (32.6)	188 (67.4)		
Education level			1.60	0.45
Illiterate	52 (36.6)	90 (63.4)		
Intermediate or lower	26 (29.5)	62 (70.5)		
Secondary or higher	21 (30)	49 (70)		
Employment			0.98	0.32
Unemployed	79 (34.5)	150 (65.5)		
Employed	20 (28.2)	51 (71.8)		
Monthly income			1.75	0.42
Low income	70 (35.4)	128 (64.6)		
Moderate income	25 (31.6)	54 (68.4)		
High income	4 (21.1)	15 (78.9)		
Presence of chronic disease			0.89	0.34
Yes	92 (33.8)	180 (66.2)		
No	7 (25)	21 (75)		
Ability to perform daily tasks			24.48	<0.001*
Dependent	23 (71.9)	9 (28.1)		
Independent	76 (28.4)	192 (71.6)		
Sleep duration			37.94	<0.001*
Normal sleep	21 (12.6)	146 (87.4)		
Abnormal sleep	77 (60.2)	51 (39.8)		
Smoking status				
Previous smokers			2.55	0.11
Yes	17 (25)	51 (75)		
No	82 (35.3)	150 (64.7)		
Current smokers			0.09	0.76
Yes	12 (35.3)	22 (64.7)		
No	87 (32.7)	179 (67.3)		
Khat chewing status				
Previous khat chewers			0.10	0.75
Yes	17 (35.4)	31 (64.6)		
No	82 (33.1)	166 (66.9)		
Current khat chewers			1.84	0.18
Yes	12 (25)	36 (75)		
No	87 (35.1)	161 (64.9)		

The results of multivariate logistic regression analysis are shown in Table 3. 

**Table 3:** Multiple logistic regression of depression among study participants. Hosmer-Lemeshow goodness-of-fit test *χ*² = 3.932. *P* = 0.863. ^*^Statistical significant result: *P* ≤ 0.05. OR, odds ratio; CI, confidence interval; Ref., reference group; NA, not applicable

Variables	OR (CI)	*P*-value
Age	1.01 (0.96 - 1.06)	0.85
Gender		
Male	0.57 (0.19-1.70)	0.31
Female	1 (Ref.)	NA
Nationality		
Saudi	0.96 (0.35-2.70)	0.95
Non-Saudi	1 (Ref.)	NA
Marital status		
Widowed and divorced	0.65 (0.22-1.93)	0.44
Married	1.17 (0.41-3.39)	0.77
Single	1 (Ref.)	NA
Residence		
Urban	1.17 (0.20-6.93)	0.86
Rural	1 (Ref.)	NA
Living condition		
Living alone	0.98 (0.28-3.37)	0.97
Living with others	1 (Ref.)	NA
Education level		
Illiterate	1.67 (0.57-5.00)	0.36
Intermediates or lower	1.65 (0.56-4.87)	0.36
Secondary or higher	1 (Ref.)	NA
Employment		
Employed	0.81 (0.36-1.79)	0.60
Unemployed	1 (Ref.)	NA
Monthly income		
Low income	1.02 ( 0.19-5.61)	0.98
Moderate income	1.31 (0.28-6.10)	0.73
High income	1 (Ref.)	NA
Presence of chronic diseases		
Yes	1.16 (0.39-3.52)	0.79
No	1 (Ref.)	NA
Ability to perform daily tasks		
Dependent	4.60 (1.56-13.59)	<0.01*
Independent	1 (Ref.)	NA
Sleep duration		
Abnormal	10.90 (5.75-20.67)	<0.001*
Normal	1 (Ref.)	NA
Smoking status		
Previous smoker		
Yes	0.75 (0.22-2.52)	0.64
No	1 (Ref.)	NA
Current smoker		
Yes	2.06 (0.54-7.84)	0.29
No	1 (Ref.)	NA
Khat chewing status		
Previous Khat chewers		
Yes	2.69 (0.75-9.66)	0.13
No	1 (Ref.)	NA
Current khat chewers		
Yes	0.94 (0.24-3.63)	0.92
No	1 (Ref.)	NA

The most important independent predictors of depression among our sample were dependency in performing daily tasks (odds ratio [OR] = 4.60; *P* < 0.01) and abnormal sleep duration (OR = 10.90; *P* < 0.001). 

## Discussion

This study assessed the prevalence of depression among older adults 60 years and above who attended the PHCCs in Jizan City, Saudi Arabia. We found that one-third of the study population was depressed; half had mild depression (16%), while the other half (17%) suffered from moderate-to-moderately severe depression. One review showed that almost 15% of older adults were affected by clinical depression (moderate to severe), which was very close to us [22]. One study in Malaysia revealed that the prevalence of depression among older people was 18% [23].

When we look at the prevalence of depression in our study, we found it lower than what had been recorded in different cities inside the Kingdom of Saudi Arabia (KSA). Four studies in other geographical areas inside KSA (Eastern Region, Altaif, Abha, and Al Madinah Al Munawwarah) showed that 42.4%, 43.2%, 63.7%, and 96.7% of study participants were depressed, respectively [24-27]. By looking at the neighboring countries, we found that the percentages of depression among older adults in Oman, Egypt, and Bahrain were 16.9%, 44.4%, and 50.6%, respectively [28-30]. This variation could be linked to the differences in sociodemographic characteristics and the use of various instrument tools [31-34]. Moreover, the lower depression score among older adults in Jizan City (33%) might be related to the strong bonds and support from family members. Many studies reveal an inverse relationship between depression and social support [35,36].

Interestingly, this study revealed an association between female gender and depression, with two-thirds of female participants and one-third of males experiencing depressive symptoms. Similar associations were reported in the literature [31,37,38]. This could happen secondary to many physiological changes during their lifetime. Also, females are more liable to harmful emotional and psychological illnesses than males [39,40].

In this study, the older adults with marital separation, such as divorced or widowed, had more depressive symptoms than married or single groups. Lacking support from the spouse and losing a loved one are known to be associated with depression [31,41]. Also, marital separation can lead to financial difficulties, and this financial stress can contribute to depression [42]. The same finding was found in other national and international studies [33,43,44].

In this study, individuals who require assistance from others to perform their daily tasks are considered dependent. From a medical perspective, we understand the dependency of older adults as a state of chronic or temporary functional disability that leads to the inability to do tasks that could be previously performed alone [45,46]. One significant independent risk factor for depression in this study was dependency. We found that people who were dependent on others and faced difficulties in doing their daily activities were more depressed than those who were not (OR = 4.60). Many studies showed an association between dependency, disability, and depression [26,47,48]. This might be because individuals with disabilities may have limited mobility or difficulty leaving home, leading to social isolation and depression [49,50]. Moreover, people with disabilities experience chronic pain that can negatively impact their quality of life and psychological status, reducing independence by making everyday tasks more challenging [51, 52].

Another significant independent risk factor of depression among older adults identified in this study was abnormal sleep duration (OR = 10.90). We considered normal sleep duration to be seven to nine hours for individuals aged 60 to 64 years and seven to eight hours for those aged 65 years and above [53,54]. People with sleep disturbances were affected by depression. Similar studies conducted in China and Japan showed an association between depression and sleeping problems [55-57]. Older adults with sleeping disturbances may be more susceptible to depression due to a combination of factors related to psychological stressors such as financial difficulties and physical health. Many older adults have chronic conditions such as chronic pain and breathing problems that can impact their sleep [58-60].

## Conclusions

Depression among older people is a prevalent health issue, with females experiencing higher levels of depression compared to males. The most important risk factors were negative marital issues, dependency, and sleep problems. More research on mental problems among this specific age group is needed.

Furthermore, encouraging older adults to remain socially engaged through activities they enjoy, such as volunteering and hobbies, can provide a sense of purpose and increase feelings of satisfaction and fulfillment.
